# The business case for quality: estimating lives saved and harms avoided in a value-based purchasing model

**DOI:** 10.1093/haschl/qxae052

**Published:** 2024-04-30

**Authors:** Peter Amico, Elizabeth E Drye, Peter Lee, Carolee Lantigua, Dana Gelb Safran

**Affiliations:** Amico Consulting LLC, Orlando, FL 32806, United States; National Quality Forum, Washington, DC 20005, United States; Stanford University Clinical Excellence Research Center, Palo Alto, CA 94304, United States; National Quality Forum, Washington, DC 20005, United States; National Quality Forum, Washington, DC 20005, United States

**Keywords:** quality measurement, health outcomes, population health, quality of care

## Abstract

Ever-increasing concern about the cost and burden of quality measurement and reporting raises the question: How much do patients benefit from provider arrangements that incentivize performance improvements? We used national performance data to estimate the benefits in terms of lives saved and harms avoided if US health plans improved performance on 2 widely used quality measures: blood pressure control and colorectal cancer screening. We modeled potential results both in California Marketplace plans, where a value-based purchasing initiative incentivizes improvement, and for the US population across 4 market segments (Medicare, Medicaid, Marketplace, commercial). The results indicate that if the lower-performing health plans improve to 66th percentile benchmark scores, it would decrease annual hypertension and colorectal cancer deaths by approximately 7% and 2%, respectively. These analyses highlight the value of assessing performance accountability initiatives for their potential lives saved and harms avoided, as well as their costs and efforts.

## Introduction

Quality measures are important for improving population health and the quality, safety, equity, and affordability of health care. Yet, providers who bear the cost and burden of reporting are often skeptical of the benefit. While the costs of measurement activities have been estimated,^1-4^ the benefits of improving performance on measures are largely unknown. To address this gap, we sought to estimate the benefits of performance improvement for 2 quality measures: colorectal cancer screening and blood pressure control among hypertensive patients.^5^ Using readily available data and peer-reviewed literature, we estimated the lives saved and harms averted if performance improved nationally in 4 market segments—Medicare, Medicaid, Marketplace, and commercial plans—and among the plans in the California Marketplace. The Covered California’s Quality Transformation Initiative (QTI), a value-based purchasing initiative that seeks to improve quality by imposing financial penalties on health plans performing below the 66th percentile on a core set of measures, was the genesis of our effort. The QTI incentivizes improvement in targeted clinical areas, including childhood immunizations, diabetes, colorectal cancer screening, and blood pressure control for those with hypertension.

After QTI's launch, California's 2 other major public purchasers (CalPERS and Medi-Cal) joined the effort. Collectively constituting 43% of California's insured population,^6,7^ these 3 purchasers are using aligned measures and performance targets. The 3 purchasers report that their contracted health plans have accepted these new terms and believe they can meet these targets as incentives increase over 3 to 5 years.

To assess the potential population benefits of QTI, we modeled the potential lives saved and harms avoided if California Marketplace health plans performing below the 66th percentile improved to that benchmark, and if the same occurred nationally across all 4 market segments. We also tested the sensitivity of results to more incremental health plan improvements and different distributions of market share in California and nationwide.

## Data and methods

For this analysis we used data from the Centers for Medicare and Medicaid Services (CMS), the State Health Access Data Assistance Center (SHADAC), the Kaiser Family Foundation (KFF), and the National Committee for Quality Assurance (NCQA). Data sources are further detailed in the Methods Appendix.

We estimated the population health benefits of improved performance on 2 measures: colorectal cancer screening (CBE #0034) and controlling high blood pressure (CBE #0018). We focused on these 2 measures because the other 2 California Marketplace measures—glycated hemoglobin (HbA1c) control among diabetics and childhood immunization status—lacked adequate data and information to enable modeling.

We modeled the incremental population health benefits for the California Marketplace and nationally across 4 market segments representing all insured populations (Medicare, Medicaid, Marketplace, and commercial) (see Methods Appendix).

Primary analyses estimated the incremental lives saved and harms avoided if performance improved to different benchmarks—the 66th and 90th percentiles. This approach allowed us to estimate the benefits in a scenario in which QTI was fully successful (all health plan performance elevated to 66th percentile or higher) and if a similar policy approach across purchasers nationally was successful. For the 1 business line for which we had plan-specific enrollment (California Marketplace), we tested the sensitivity of our results to a model with more incremental health plan performance improvement where plans below 66th percentile performance each improved only to the next performance decile.

In order to calculate the additional number of people in the California Marketplace plans and nationally who would achieve the measured clinical status, and translate that to population health benefits for each measure, we undertook the following steps. First, we categorized plans into 5 market segments: California Marketplace plans and 4 national segments (Medicare, Medicaid, Marketplace, and commercial). For Medicare and Medicaid, we used benchmarks for those in managed-care plans but extrapolated to the full population. Second, we estimated the proportion of participants in each segment who would move into the measure numerator with improved performance, using California Marketplace and national 2019 health plan measurement year performance benchmarks at the 66th and 90th percentiles. We modeled the benefits of plans below the 66th percentile benchmark achieving the 66th percentile performance, and similarly, those below the 90th percentile performing at that benchmark. Conservatively, we assumed plans between the reported percentiles (25th, 50th, 66th, 75th, and 90th) were at the next highest percentile (eg, we assumed all plans between the 1st and 25th percentile were at the 25th percentile). We also tested the sensitivity of this assumption by modeling improvement to the next decile of performance for each measure in the California Marketplace where data were available. Third, we estimated the population in the California Marketplace and nationally in each segment in the 2 measure denominators, using California and national enrollment information distributed by age^8-12^ and, for hypertension, national disease prevalence rates by age groups.^13,14^ Fourth, we calculated the additional patients who would move into the numerator (screened for colorectal cancer or who had controlled hypertension) with improved plan performance. We assumed that plan market share was evenly distributed across the percentiles of performance due to unavailable data. We tested this assumption by performing a sensitivity analysis using California Marketplace data, the 1 market segment for which we had plan-specific enrollment data.

Finally, to estimate lives saved or harms avoided for the marginal plan members screened or who achieved hypertension control, we estimated the “number needed to treat” (NNT) to avert death for each intervention and applied it to the number of additional people in the numerator. Consulting with clinical and methods experts, we derived the NNTs from the most relevant literature. To derive the NNT for colorectal cancer screening, we selected the 2021 simulated cohort study informing the US Preventive Services Task Force (USPTF) recommendations.^15^ For hypertension, we derived the NNT from the meta-analysis of risk reduction associated with blood pressure treatment that best corresponded to the measure's target blood pressure.^16^

To estimate the health benefits of colorectal cancer screening, because the USPTF recommendations and the corresponding measure numerator include multiple screening approaches, we used the health benefits associated with colonoscopy; results for other guideline-based screening modalities had similar estimated risk reductions (eg, biannual fecal immunochemical testing). The USPTF study estimated colonoscopy screening per guidelines among 50- to 75-year-olds would avert 27 deaths per 1000 individuals (NNT = 37) over their lifetimes, on average, among 3 microsimulation models.^17^ We converted the estimate of lives saved into an annual estimate using remaining life expectancy distributions, estimating 22 years of average remaining life expectancy after screening is initiated at age 50.^18^

**Figure 1.**
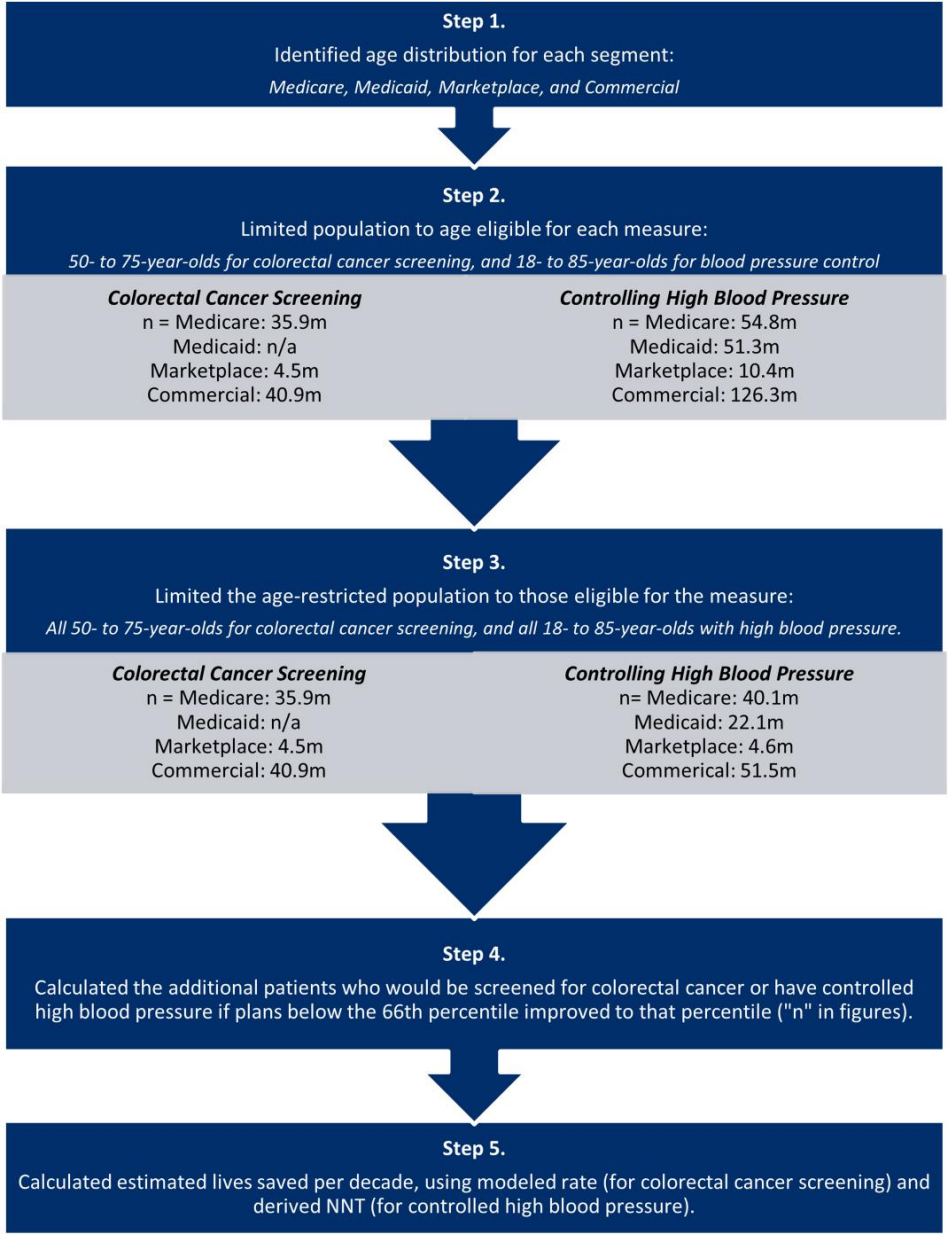
Quality-impact model methodology flowchart for national estimates. Source: Authors' depiction of the steps to estimate lives saved. Abbreviations: n/a, not available; NNT, number needed to treat.

To estimate the hypertension-control health benefits, with input from experts, we selected the primary prevention arm of a meta-analysis of randomized therapeutic trials by Brunström and Carlberg^19^ as the study best aligned with the hypertension measure's goal. We assumed that the clinical benefits of treating patients with a systolic blood pressure (SBP) of 140 to 159 mm Hg to below 140 mm Hg roughly correlated with the benefits of the treatments provided in the study for this group (39.9% women and 60.1% men; mean age, 63.6 years; average SBP at study outset between 140 and 159 mm Hg). In this cohort, the median follow-up period was 4 years, and SBP decreased by an average of 6.6 mm Hg. We derived the NNT to prevent 1 death from the study's estimated absolute risk reduction for all-cause mortality over the 4-year study. Figure 1 graphically depicts the estimation process.

## Results

Across all segments, health plan performance variation from the 25th to 90th percentiles ranged from 47% to 83% (Table 1). Marketplace plans showed the most variation and Medicare the least.

**Table 1.** Measure scores for colorectal cancer screening and controlling high blood pressure at the 66th percentile performance by market segment.

	Percentiles									
	Colorectal cancer screening measure health plan distribution score					Controlled high blood pressure measure health plan distribution score				
	25th	50th	66th	75th	90th	25th	50th	66th	75th	90th
Medicare	67	74	78	80	83	65	71	74	76	81
Medicaid*	—	—	—	—	—	53	61	65	67	72
Marketplace	47	55	61	63	69	54	62	66	70	75
Commercial	57	62	66	68	74	52	60	64	67	74
California Marketplace	47	54	57	60	65	57	65	66	68	76

*Medicaid data not available for Colorectal Cancer Screening measure.

Source: Authors' analysis of data from Medicare Part C and D performance data and the Marketplace Quality Rating System and National Committee for Quality Assurance (NCQA) Quality Compass, 2020. Data were not available for Medicaid.

### Colorectal cancer screening

**Figure 2.**
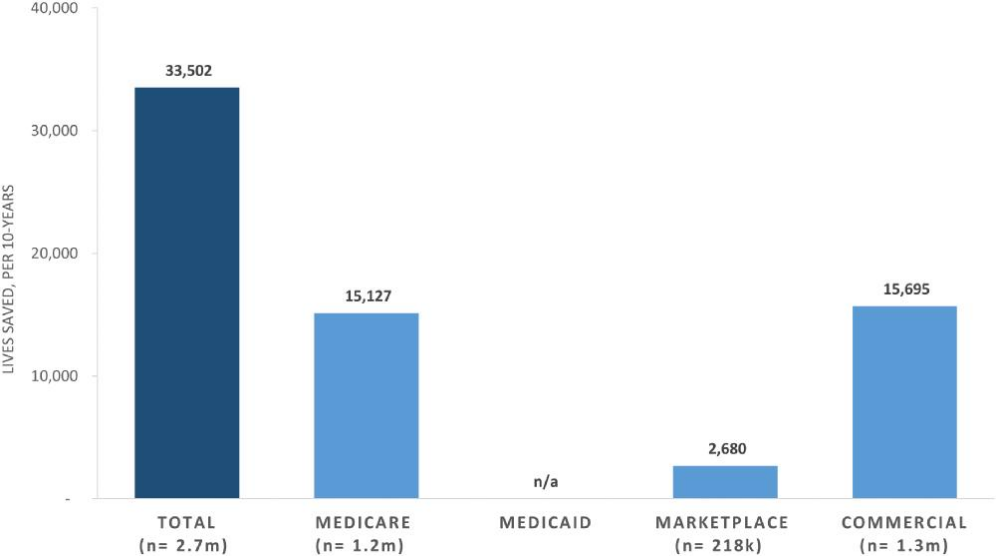
Additional lives saved, per decade, if all national plans improve colorectal cancer screening measure performance to the 66th percentile, by market segment. Source: Authors' analysis of data from Medicare Part C and D performance data and the marketplace quality rating system and NCQA quality compass, 2020. Abbreviations: n/a, not available; NCQA, National Committee for Quality Assurance.

Improving all plans in the lower two-thirds of the distribution to the 66th percentile within each segment increased the people in the numerator as follows: California Marketplace, 20 865; Marketplace, 217 638; commercial, 1 274 794; and Medicare, 1 228 663. (We were unable to estimate the affected Medicaid population for this measure because plan performance data were not available.) Applying the colorectal cancer screening NNT to the additional screened patients, we estimated that, by reaching the 66th percentile of performance (Figure 2), 33 502 additional lives would be saved each decade across all 3 markets (Medicare, 15 127; Marketplace, 2680; commercial, 15 695), with 257 lives saved in the California Marketplace. If all plans performed as well as the 90th percentile plan nationally, 85 432 total lives would be saved over a decade (Medicare, 46 027; Marketplace, 5992; commercial, 33 412), with 679 lives saved in the California Marketplace.

When we tested the sensitivity of the results to different assumptions about market share and performance improvement to the next decile, lives saved changed by +18% (improvement to the next decile, retaining the assumption of even population distribution) to −2% (improvement to the next decile, actual population distribution in California Market plans). Additional details are provided in the Methods Appendix.

### Controlling high blood pressure

**Figure 3.**
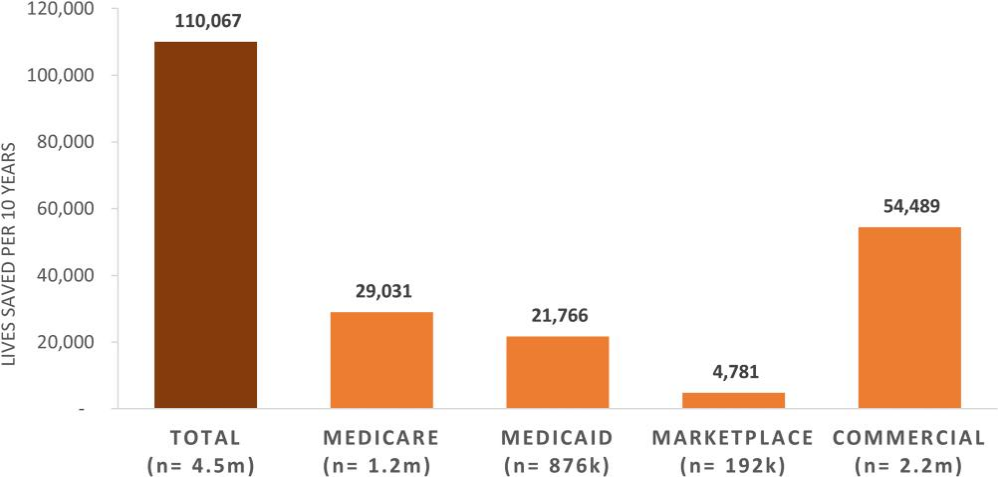
Lives saved, per decade, if all US plans improve hypertension measure performance to the 66th percentile, by market segment. Source: Authors' analysis of data from Medicare Part C and D performance data and the marketplace quality rating system and National Committee for Quality Assurance (NCQA) quality compass, 2020.

Raising all plans to the 66th percentile performance within each segment increased the total number of people in the measure numerator (ie, with SBP <140 mm Hg and diastolic blood pressure [DBP] <90 mm Hg) as follows: California Marketplace, 13 880; Medicare, 1 168 105; Medicaid, 875 763; Marketplace, 192 372; and commercial, 2 192 477. For hypertension, no study in the literature examined the benefits of achieving the blood pressure target specified in the measure numerator (the proportion of patients with SBP <140 mm Hg and DBP <90 mm Hg). The calculated NNT to prevent 1 death was 101. Applying the NNT to the marginal patients with controlled hypertension, we estimated that sustained health plan performance over a decade at the 66th percentile would save 110 067 lives across all 4 market segments nationally (Figure 3), with 330 lives saved in the California Marketplace. Finally, lives saved across markets if all plans performed as well as the 90th percentile nationally were 280 487 total (Medicare, 80 080; Medicaid, 51 760; Marketplace, 12 260; commercial, 140 060), with 1320 lives saved in the California Marketplace. Of important note, while the age-based prevalence of hypertension is higher in Medicare (73% vs 41%), the overall lives saved are lower than commercial due to the larger commercial insurance population (169 million vs 61 million), the more compressed performance range among plans, and the higher baseline performance of Medicare plans. In addition to mortality, using the same study, we estimated 137 164 averted major cardiovascular events with increased performance at the 66th percentile, and 335 006 at the 90th percentile over 10 years across all markets.

When we tested the sensitivity of the results to different assumptions about market share and performance improvement to the next decile, lives saved changed by +6% (improvement to the next decile, retaining assumption of even population distribution) to +3% (improvement to the next decile, actual population distribution in California Marketplace plans). Additional details are provided in the Methods Appendix.

## Discussion

Using readily available data and performance benchmarks, we estimated lives saved and harms avoided if plans improve performance on 2 measures widely used in performance accountability programs. Our question was spurred by interest in the potential health impact of a California value-based purchasing program (QTI), but it has broader relevance. We modeled the results for California Marketplace plans and nationally. We focus the discussion here on the national results for ease of interpretation, although the California results are similar in scale relative to the California population. Nationally, we estimated that improving performance to the 66th percentile would save 34 000 and 110 000 lives for colorectal cancer screening and blood pressure control, respectively, if improvements were sustained over 10 years.

**Figure 4.**
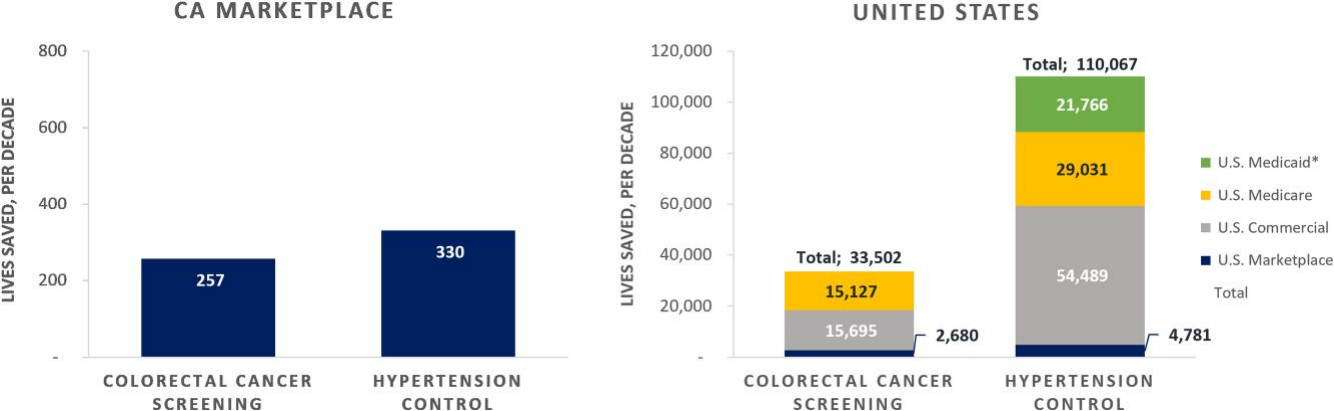
Lives saved, over 10 years, if California (CA) marketplace plans and all plans nationally improve measure performance to the 66th percentile, by market segment. Source: Authors' analysis of data from Medicare Part C and D performance data and the marketplace quality rating system and National Committee for Quality Assurance (NCQA) quality compass, 2020. *Medicaid data not available for Colorectal Cancer Screening measure.

We used the 66th percentile (ranging from plan measure performance of 57% to 78% across the 2 measures and 5 segments) (Figure 4) as our primary target because evidence suggests that it is achievable for most plans, even those with populations that have higher disease burdens and/or contributing social risk factors. Improving all plans to the 90th percentile performance is more aspirational.

It is possible that the resources required for improvement are greater or different for plans serving different patient populations—and notably, those with higher social risk. Some have suggested that an optimal policy approach is to offer higher financial rewards (or lower financial penalties) to those serving a more vulnerable population, while continuing to hold all accountable to the same performance standard on the measures.^20^ This approach aims to invest in achieving greater health equity as opposed to setting lower performance standards for those serving a more vulnerable population.

These numbers may seem relatively small, considering that these 2 conditions are leading causes of death nationally. Colorectal cancer and hypertension cause an estimated 51 869 and 691 095 deaths in the United States annually.^21,22^ However, the marginal contribution of improving plan performance to reducing annual US condition-specific deaths is not trivial; raising plans to 66th percentile performance would lower colorectal cancer and hypertension deaths annually by 6.9% and 1.6%, respectively. Raising performance to the 90th percentile would reduce annual colorectal cancer and hypertension deaths by 17.5% and 4.0%, respectively.

Health plans and other capitated payment structures (eg, accountable care organizations) are increasingly investing in innovative, person-centered care to improve health and wellness across all lines of business and populations. And the benefit of getting there, as noted above, is substantial, and would lower disparities in care, given that historically marginalized populations and patients with social risk factors often bear the largest gaps in preventive care.^23^ In California, 3 purchasers (Covered California, CalPERS, and Medi-Cal) now have contract terms with the expectation of plans achieving the 66th percentile of performance. Covered California has already seen significant improvement for several underperforming plans.

This study aims to start building the evidence that will inform selection and drive consensus on alignment of measures across payers and programs to optimize return on investment by better understanding and communicating the value in lives saved and harms avoided by improving performance. To lower provider burden and increase impact, policymakers and payers are working to speed adoption of aligned measure sets that focus on measures that could have the greatest impact on outcomes and equity. In California, CalPERS, Covered California, and the state Medi-Cal program chose to focus on a handful of high-impact measures, including the 2 we studied. Quality leaders across 3 CMS centers similarly have identified a Universal Foundation with only 11 adult measures, including the colorectal cancer screening and controlling high blood pressure measures. More evidence is needed to inform these critical alignment efforts; nevertheless, creating parsimonious, aligned measure sets based on their potential to save lives and avoid harm is a worthy approach. Robust estimates of costs and benefits are also needed to inform decisions about the merits of the programs designed to incentivize performance improvement.

### Limitations

Our approach relied on estimating benefits using performance distributions, readily available population data, and a per-person estimate of mortality reduction benefit derived from clinical literature. This was inherently limiting. For example, we could not estimate the benefits of the diabetes measure in the California Marketplace—the proportion of patients with diabetes and with HbA1c levels below 8%—because no study in the literature was suitable for estimating the risk reduction and related NNT. Available studies have limited alignment with the measure's structure because the patients studied largely already have met the measure's target HbA1c levels, and the life-saving benefits reported accrue, in part, through mechanisms independent of the glucose-lowering achieved over long time periods.^24-27^ Furthermore, health plan performance distribution data are proprietary, and sharing them is not allowed due to the perception of competition in some contexts. More nuanced modeling of the benefits of incentivizing performance on quality measures is needed. We are pleased that the American Medical Association is pursuing such an effort for hypertension as part of its Measure Accurately, Act Rapidly, and Partner with Patients (MAP) Protocol.^28^

Additional work will also need to be done to assess the impact on providers serving high-need populations and supporting their success. The 3 public purchasers in California all have identified this as an area for ongoing monitoring and Medi-Cal has added an adjustment factor to their quality withhold based on the Healthy Places Index. At the federal level, the Medicare Shared Savings Program recently added Advance Investment Payments for participating qualified rural and underserved communities to support standing up the infrastructure and improvements needed to succeed in achieving savings and health benefits.^29,30^

Our pragmatic approach to estimating the health benefits of incentivizing performance on the 2 measures has several further limitations, as follows:

1. To simplify our calculations and provide for cross-measure comparisons, we only estimated a subset of the known benefits; for both measures, we estimated lives saved and for the hypertension measure, major cardiac events (including deaths) averted. However, colorectal cancer screening and blood pressure control among hypertensive patients lead to many other benefits, including a lower incidence of and better outcomes for patients with colorectal cancer,^31,32^ and fewer nonfatal, nonmajor cardiovascular events, such as kidney failure, vision loss, and general disability.^33-35^
2. To calculate the NNT for blood pressure control, we relied on a meta-analysis answering a question related to, but not fully aligned with, the blood pressure measure structure (ie, it considered only SBP), which could have under- or overstated the risk reduction. In addition, the estimated effect sizes from the literature were not specific to the 4 market segments.
3. We used 6 discrete percentile scores rather than the continuous distributions of health plan performance, and conservatively assumed plans between the percentiles were already all at the higher percentile of performance, understating the benefits of improvement. We also assumed that patients were distributed equally across the performance percentiles of the health plans, possibly overstating benefits, given that larger plans perform better on quality measures on average, likely due to more robust infrastructure and resourcing.
4. The relatively small potential improvement benefits in Medicare reflects the higher health plan performance across the distribution among Medicare plans compared with other market segments, which is consistent with the significant financial incentives attached to these measures for Medicare Advantage plans, and may not generalize to traditional Medicare, where opportunities for improvement may be significantly greater.^36^
5. We may be understating the potential impact of QTI's incentives by estimating only the marginal benefits associated with improving plans below the 66th percentile to that level; QTI's incentive structure could be an important driver for delivering the targeted care and improving outcomes. Since lowering blood pressure reduces mortality risk across a wide range of blood pressures,^16,37^ health plans' efforts to improve care incentivized by quality measures likely benefit many more patients than those we counted (eg, those newly diagnosed through better screening programs for hypertension, those whose blood pressures were already lower than 140/90 mm Hg and whose blood pressure was reduced through improved care, or those whose blood pressure improved but remained above the 140/90-mm Hg thresholds). Finally, we used national age, prevalence, and performance distributions disregarding regional variation in each, introducing substantial uncertainty.
6. The lack of available data on national plan enrollment by performance could bias the results either upward or downward depending on the distribution of enrollment by plan performance. For example, the fact that 33% and 16% of the California Marketplace plan population for the hypertension control and colorectal screening measures, respectively, were in the lowest-performing plans likely accounts for the finding in sensitivity analyses of an increased impact on lives saved for hypertension control (3%) and a smaller impact on lives saved for colorectal cancer screening (−2%) when the actual population distribution was accounted for in our modeling.

## Conclusion

With the quality-measurement enterprise increasingly criticized for the burden and costs for providers,^38^ the lack of data quantifying the value of performance improvement in terms of lives saved and harms avoided is a critical gap for policymakers, purchasers, health plans, and consumers. This information is critical to appropriately direct resources to performance accountability initiatives that yield benefits worthy of their costs. This model illustrates how this can be accomplished and the significant benefits associated with 2 measures that are widely used by public and private-sector performance-based payment programs.

## Supplementary Material

qxae052_Supplementary_Data
