# Supplementary material for: The business case for quality: estimating lives saved and harms avoided in a value-based purchasing model
Source: Health Aff Sch. 2024 Apr 30;2(5):qxae052. doi: 10.1093/haschl/qxae052 (PMC11098439; doi:10.1093/haschl/qxae052)
Supplement: qxae052_Supplementary_Data [file qxae052_supplementary_data.zip › Quality Impact Model - Methods Appendix_final.docx]

## The Business Case for Quality: Evaluating lives saved and harms avoided in a value-based purchasing model – Methods Appendix

This document provides detailed information about the estimated health impacts reported in the submitted manuscript. As the manuscript notes, the overarching goal was to use available population data, health plan performance data, and estimated effects from the literature to compute estimated health impacts for relevant market segments. Here are the steps we took to estimate health impacts:

1. Identified relevant meta-analyses and/or controlled clinical trials and selected the study that best aligned with the quality measure numerator and the denominator population, and that was consistent with current treatment practices. The health gains estimated in the selected study for each measure are the basis for the health impacts reported for the measured populations.
2. Limited studies to those that enabled us to estimate a number needed to treat (NNT).
3. Selected outcomes from the study results – mortality and other major events – that were most clinically salient.
4. For hypertension control, the absolute risk was not available for both the control and the treatment group. Therefore, we calculated the absolute risk for the treatment group, RateT= (events/participants), and then calculated the absolute risk for the control group, RateC = (RateT*[1/Relative Risk]).
5. When there were two or more screening modalities with similar results (colorectal cancer screening), we selected one modality (colonoscopy) in consultation with clinical experts. For colonoscopy outcomes, deaths averted was taken directly from the modeled estimated population benefit of screening published in the literature. It provided deaths averted per 1,000. We divided 1,000 by the deaths averted to get the NNT. The NNT methods are summarized in Table 1 below.
6. All outcomes were adjusted for the median length of follow-up to present an annualized outcomes averted result. Median follow-up was four years for hypertension. For colonoscopy screening, we calculated the average remaining life expectancy of those eligible for screening (21.93 years) and divided the effect size by that amount to get an annual expected lives saved. Life expectancy was derived using the age/gender weighted average remaining life expectancy from the 2019 Social Security Association (SSA) life tables for the 2021 Trustees Report.^^[[1]](#footnote-1)^^ The median follow-up periods are summarized in Table 1 below.

**Table 1: Number Needed to Treat (NNT) Methods and Median Follow-Up Periods**

|  | Hypertension Control | Colonoscopy Screening |
| --- | --- | --- |
| NNT Method | 1/(RateC-RateT)  RateT calculated as events/participants.  RateC= RateT*(1/Relative Risk) | Deaths averted provided in literature converted to NNT= 1,000/deaths averted per 1,000 |
| Median Follow-up | 4 years | 21.93 years remaining average life expectancy |

1. We selected and reported one or more outcomes for each quality measure, focusing on those of greatest clinical impact. The outcomes are listed in Table 2 below.

**Table 2: Clinical Outcomes for the Two Quality Measures**

|  | Hypertension Control | Colorectal Cancer Screening  (specifically, colonoscopy) |
| --- | --- | --- |
| Outcomes | ***All-Cause Mortality*** (140-159 mm Hg and >160 mm Hg), ***Major Cardiovascular events*** (140-159 mm Hg and >160 mm Hg) | ***All-Cause Mortality*** |

1. We pulled the available age distributions for the four major national market segments: Commercial^[[2]](#footnote-2)^, Marketplace^[[3]](#footnote-3)^, Medicare,^[[4]](#footnote-4)^ and Medicaid^[[5]](#footnote-5)^ populations as well as the California Marketplace. We then calculated the total number of people potentially eligible for each measure based on the measure specifications (colorectal cancer screening [ages 50 to 75]; hypertension control [ages 18 to 85]). When age distributions were not specific enough for the 65+ aged segment of the measure population, we applied the proportion of Medicare patients over age 65 to estimate the proportion of people in the eligible age range. The measures have other limited exclusions which we did not account for (e.g., people in hospice). The age groups are presented below in Table 3.

**Table 3: Eligible Population by Payer Type**

|  | Total | Hypertension  Control | Colorectal Cancer Screening |
| --- | --- | --- | --- |
| Eligible Population |  | 18 to 85 years old | 50 to 75 years old |
| Commercial (2019) | 168,899,543 | 126,300,656 | 40,858,780 |
| Marketplace (2019) | 11,444,141 | 10,440,352 | 4,451,686 |
| Medicare (2019) | 61,514,510 | 54,816,723 | 35,873,367 |
| Medicaid (2019) | 87,868,100 | 51,340,277 | N/A |
| California Marketplace (2019) | 1,513,883 | 1,408,037 | 604,772 |

Note: California Marketplace estimates were provided from the same Kaiser Family Foundation source as the national estimates.

1. After calculating the population eligible for the measure by age, we further limited the population to those who qualified for each based on their diagnosis.
   1. To estimate those eligible for the hypertension control measure, we used the prevalence, stratified by age band: ages 18 to 44 (26.4%), ages 45 to 64 (43.9%), and ages 65+ (77.1%).^[[6]](#footnote-6)^ We multiplied the national eligible population by the approximate hypertension prevalence rates for each age band in the measure denominator (18 to 85 years old) to estimate the total population in the measure by market segment. California’s Marketplace prevalence was estimated to be 14% lower than the national average using the Behavioral Risk Factor Surveillance System self-reported hypertension measure compared to the national self-reported hypertension measure.^^[[7]](#footnote-7)^^
   2. To estimate those eligible for the colonoscopy screening measure, we did not make any restrictions other than to limit the population to those aged 50 to 75 years as required by the measure. Table 4 below shows the total eligible population that could benefit from quality improvement.

**Table 4: Total Eligible Population That Could Benefit from Quality Improvement**

| Market Segment | Commercial | Marketplace | Medicaid | Medicare | California Marketplace |
| --- | --- | --- | --- | --- | --- |
| Hypertension Control, eligible population  (ages 18 to 85) with uncontrolled hypertension | 51,465,884 | 4,613,538 | 22,115,238 | 40,141,056 | 560,241 |
| Colorectal cancer screening (colonoscopy), eligible population  (ages 50 to 75) | 40,858,780 | N/A | 12,210,340 | 35,873,367 | 604,772 |

Note: California’s hypertension prevalence rate was estimated to be 14% lower than the national rate.

1. Once we calculated the total eligible population that could benefit from the quality intervention, we then estimated the proportion of participants in each segment who would move into the measure numerator with improved performance, using California Marketplace and national 2019 health plan performance benchmarks at the 25th, 50th, 66th, 75th, and 90th percentiles. For example, we modeled the benefits of plans below the 66th percentile benchmark achieving the 66th percentile performance, and similarly those below the 90th percentile performing at that benchmark. Conservatively, we assumed plans between the reported percentiles were at the next highest percentile (e.g., we assumed all plans between the 1st and 25th percentile were at the 25th). For Marketplace plans, we used health plan performance data from the CMS Quality Rating System (QRS) which is a reporting structure for marketplace plans. ^[[8]](#footnote-8)^ Performance data were used from plan year 2021, which used measurement data from 2019 before the COVID-19 pandemic. For Medicare hypertension performance, we used Part C and D measurement performance data from 2019. For all other measures and market segments, we obtained performance data from 2019 measurement year from NCQA’ s Quality Compass.^[[9]](#footnote-9)^ All performance data were based on HEDIS measures which require a sample size of 411 patients abstracted from medical record review in order to be reported.^[[10]](#footnote-10)^
2. To quantify the impact of improved measure performance, we multiplied the eligible population that could benefit from the quality intervention by the change in quality measure performance at the various plan percentiles in the setting of improved health plan performance. To be conservative, we assumed health plans are performing at the highest percentile for a given percentile range. For example, we assumed that plans between the 0 and 25th percentile are all performing at the 25th percentile. Thus, plans at the 25^th^ percentile of performance for hypertension control (51.89%) and those at the 90^th^ percentile of performance (72.26%) would improve by 72.26% - 51.89%= 20.37%. That difference in control is then multiplied by the total eligible population and then divided by 1/additional population, in this case 1/.25= 4. This was done because only 25% of the impacted population will benefit. Table 5 below shows the different population divisors used for each plan performance cohort.

**Table 5: Population Divisors**

| Plan Performance Cohort | Percent of the Population | Cohort Divisor |
| --- | --- | --- |
| 0-25^th^ percentile | 25% | 1/.25= 4 |
| >25^th^-50^th^ percentile | 25% | 1/.25= 4 |
| >50^th^- 66^th^ percentile | 16% | 1/.16=6.25 |
| >66^th^ – 75^th^ percentile | 9% | 1/.09= 11.11 |

1. For cohorts starting at the 50th, 66^th^, and 75^th^ percentile, we assumed different cohorts of control. For example, moving from the 75^th^ to the 90^th^ percentile of control assumed that 25% of the population was at the 25^th^ percentile of control, that the next 25% of the population was at the 50^th^ percentile of control, the next 16% of the population was at the 66^th^ percentile of control, and the next 9% were at the 75^th^ percentile of control. We also assumed that the population was evenly distributed by plan performance. Therefore, we assumed that 25% of the population was represented by the 25^th^ percentile of plan performance. To account for this, each cohort was divided by the cohort divisor to represent only the percent of the population impacted in the cohort. An additional conservative element of the methodology is that no improvement is calculated for the cohort just below the “benchmark” (e.g., in assessing performance improvement to the 90^th^ percentile, there is no improvement value assessed for the range for the 76^th^ to 90^th^ percentile, see Table 6). Table 6 below shows the cohorts included in the calculation of the marginal population that would benefit from the improved performance for the specified quality measure.

**Table 6: Cohorts Included in the Performance Calculation**

|  | Cohorts Included in Calculation |
| --- | --- |
| All plans to 66^th^ Percentile | 0-25^th^ to 66^th^ +>25^th^- 50^th^ to 66^th^ + >50^th^ – 66^th^ to 66^th^ |
| All plans to 90^th^ Percentile | 0-25^th^ to 90^th^ +>25^th^- 50^th^ to 90^th^ + >50^th^ – 66^th^ to 90^th^ + >66^th^-75^th^ to 90^th^ + 76^th^-90^th^ to 90^th^ |

1. We estimated the increase in the number of patients who would be successful “in treatment” (achieving the quality measure numerator clinical target) if plans raised their performance on the quality measures.
2. After these calculations were complete, we were left with the following:
   1. Total number of marginal patients who would be impacted by the improved health plan performance for each quality measure.
   2. The Number Needed to Treat (NNT) for each outcome (mortality, diabetes-related event, myocardial infarction, etc).
   3. The study median follow-up time/average remaining life expectancy.
3. To calculate the number of lives saved and harms averted, we divided the marginal controlled/screened population by the NNT to get an estimate of the total number of events averted given the improvement in hypertension control and colorectal cancer screening. Then we divided that by the median study follow-up time/average remaining life expectancy (colonoscopy screening) to get an annual number of events averted.
4. *We estimated two alternative scenarios for Covered California specifically because that was the only market where we had both plan level performance and market share available.*

*First, we estimated the deaths averted if each plan increased from their baseline performance to the nearest decile of performance and we assumed market share was distributed evenly across plans. Second, we estimated the deaths averted if each plan increased their baseline performance to the nearest performance decile and we used the plan’s actual market share. We estimated the sensitivity analyses using the hypertension and colorectal cancer screening measures. Due to data limitations, we only had plan market share from the Covered California Marketplace.*

*We compared these scenarios to the status quo methodology that we used in the paper, assuming that all plans increase to the 66^th^ percentile of performance and that market share is distributed evenly. For hypertension We found that the first method (1 decile improvement + equal market share) increased the deaths averted by 6% from the status quo, and the second method (1 decile improvement + actual market share) increased deaths averted by 3% from the status quo.*

**Hypertension Control Sensitivity Analysis**

|  | 25 to 66th Status Quo | Decile+ Even Market Share | Decile + Actual Market Share |
| --- | --- | --- | --- |
| Deaths Averted | 330 | 350 | 339 |
| Pct from Status Quo |  | 6% | 3% |

We analyzed the same effect using the colorectal cancer screening measure. The first method (1 decile improvement + equal market share) increased the deaths averted by 18% from the status quo, and the second method (1 decile improvement + actual market share) decreased deaths averted by 2% from the status quo.

**Colorectal Cancer Screening Sensitivity Analysis**

|  | 25 to 66th Status Quo | Decile+ Even Market Share | Decile+ Actual Market Share |
| --- | --- | --- | --- |
| Deaths Averted | 257 | 302 | 251 |
| Pct from Status Quo |  | 18% | -2% |

The primary reason for the difference from the hypertension measure is that the colorectal cancer screening measure had a smaller proportion of the population in the lowest quartile plans by performance. For hypertension control, 33 percent of the covered population were in the lowest performing quartile plans, thus the actual market share led to an increase in lives saved (+3%). Only 16 percent of the population were in the lowest performing quartile for colorectal cancer screening, thus using the actual market share led to a decrease in estimated lives saved (-2%). Using actual market share would be preferred but is not readily available.

**Percent of the Population Based on Performance Measure**

| Population by quartile of performance | Colorectal | Hypertension |
| --- | --- | --- |
| 0-25 | 16% | 33% |
| 26-50 | 21% | 14% |
| 51-66 | 12% | 4% |
| 67-75 | 13% | 5% |
| 76-100 | 38% | 44% |

1. [Social Security Administration – Period Life Table, 2019, as used in the 2021 Trustees Report](https://www.ssa.gov/oact/STATS/table4c6_2019_TR2021.html) [↑](#footnote-ref-1)
2. [State Health Compare by Shadac – Health Insurance Coverage Type](https://statehealthcompare.shadac.org/table/4/health-insurance-coverage-type-by-age#1,6/3,12,13,20,25,14,21,22,23,24,11,86/27/7,8) [↑](#footnote-ref-2)
3. [KFF – State Health Facts - Marketplace Plan Selection by Age](https://www.kff.org/health-reform/state-indicator/marketplace-plan-selection-by-age/?currentTimeframe=4&selectedRows=%7B%22wrapups%22:%7B%22united-states%22:%7B%7D%7D,%22states%22:%7B%22california%22:%7B%7D%7D%7D&sortModel=%7B%22colId%22:%22Location%22,%22sort%22:%22asc%22%7D) [↑](#footnote-ref-3)
4. [CMS – CMS Program Statistics – Medicare Total Enrollment](https://data.cms.gov/summary-statistics-on-beneficiary-enrollment/medicare-and-medicaid-reports/cms-program-statistics-medicare-total-enrollment) [↑](#footnote-ref-4)
5. [KFF](https://www.kff.org/medicaid/state-indicator/medicaid-enrollment-by-age/?dataView=1&currentTimeframe=0&selectedRows=%7B%22wrapups%22:%7B%22united-states%22:%7B%7D%7D,%22states%22:%7B%22california%22:%7B%7D%7D%7D&sortModel=%7B%22colId%22:%22Location%22,%22sort%22:%22asc%22%7D) – State Health Facts – Medicaid Enrollment by Age [↑](#footnote-ref-5)
6. [Million Hearts – Estimated Hypertension Prevalence, Treatment, and Control Among US Adults: Tables](https://millionhearts.hhs.gov/files/Estimated-Hypertension-Prevalence-tables-508.pdf) [↑](#footnote-ref-6)
7. [CDC – BRFSS Prevalence & Trends Data](https://nccd.cdc.gov/BRFSSPrevalence/rdPage.aspx?rdReport=DPH_BRFSS.ExploreByLocation&irbLocationType=States&islClass=CLASS01&islLocation=06&islTopic=TOPIC03&islYear=2021&rdRnd=98214) [↑](#footnote-ref-7)
8. [CMS – About Quality Rating Systems (QRS)](https://www.cms.gov/medicare/quality-initiatives-patient-assessment-instruments/qualityinitiativesgeninfo/aca-mqi/quality-rating-system/about-the-qrs) [↑](#footnote-ref-8)
9. NCQA, Quality Compass (2020). [↑](#footnote-ref-9)
10. https://omb.report/icr/202006-0938-017/doc/102200900 [↑](#footnote-ref-10)
